# A polymorphism in the 3' untranslated region of the gene encoding prostaglandin endoperoxide synthase 2 is not associated with an increase in breast cancer risk: a nested case-control study

**DOI:** 10.1186/bcr1635

**Published:** 2007-01-10

**Authors:** David G Cox, Julie Buring, Susan E Hankinson, David J Hunter

**Affiliations:** 1 Program in Genetic and Molecular Epidemiology, Epidemiology Department, Harvard School of Public Health, 677 Huntington Avenue, Boston, MA 02115, USA; 2 Channing Laboratory, Department of Medicine, Brigham and Women's Hospital, Harvard Medical School, 181 Longwood Avenue, Boston, MA 02115, USA; 3 Division of Preventive Medicine, Department of Medicine, Brigham and Women's Hospital, Harvard Medical School, 900 Commonwealth Avenue, Boston, MA 02215, USA; 4 Epidemiology Department, Harvard School of Public Health, 677 Huntington Avenue, Boston, MA 02115, USA

## Abstract

**Introduction:**

Prostaglandins are integral components in the cellular response to inflammation, promoting cellular proliferation and angiogenesis. The enzyme responsible for the conversion of arachidonic acid to prostaglandins in response to inflammation is prostaglandin endoperoxide synthase 2/cyclo-oxygenase 2 (PTGS2/COX2). Polymorphisms in the *PTGS2 *gene have been associated with various diseases, including inflammatory bowel disease and cancer of the lung, colorectum, and breast.

**Methods:**

We genotyped the five most common polymorphisms (rs20417, rs5277, rs20432, rs5275, and rs4648298) in the Nurses' Health Study (1,270 cases, 1,762 controls) to test the hypothesis that polymorphisms in *PTGS2 *are associated with breast cancer risk, using logistic regression analyses. The Nurses' Health Study 2 (317 cases, 634 controls) and Harvard Women's Health Study (702 cases, 703 controls) were used to further examine putative associations.

**Results:**

The rs5275 polymorphism in the 3' untranslated region of the *PTGS2 *gene was associated with a decrease in breast cancer risk. We therefore genotyped this single-nucleotide polymorphism in the Nurses' Health Study 2 and Harvard Women's Health Study. Similar results were observed in these subsequent analyses, with no statistically significant heterogeneity in risk estimates between studies. In pooled analyses, women homozygous for the T allele at rs5275 had a 20% lower risk of breast cancer than those homozygous for the C allele (odds ratio 0.80, 95% confidence interval 0.66 to 0.97).

**Conclusion:**

Although this polymorphism may be associated with a decrease in breast cancer risk among Caucasian women, we provide strong evidence that it is not associated with an increased risk of breast cancer.

## Introduction

Inflammation is thought to have a role in many diseases, including cancers [1]. Cellular response to inflammation signaling can lead to increased expression of growth factors and their receptors, which in turn can lead to cellular proliferation [2]. In addition, by-products of the inflammatory response, such as free radicals, can cause DNA damage [3-5]. Taken together, these factors suggest a strong *a priori *hypothesis that inflammation is related to the etiology of cancer.

Although there is an inflammatory type of breast cancer, this accounts for only 1 to 5% of the overall incidence of breast cancer in the United States [6]. Non-steroidal anti-inflammatory drugs have been shown to be inversely associated with risk of colorectal cancer risk [7]. However, the association between these drugs and breast cancer risk is unclear [8,9]. Nevertheless, causal relationships between prostaglandin endoperoxide synthase 2/cyclo-oxygenase 2 (PTGS2/COX2) and mammary epithelial tumorigenesis have been detected in animal models [10].

PTGS2 is a protein that is expressed as part of the acute response to inflammation [11] and is the main enzyme responsible for transforming arachidonic acid into prostaglandins. Prostaglandins in turn stimulate cell proliferation and angiogenesis. Expression of PTGS2 is tightly controlled, with basal levels low to nonexistent in most tissue types. In addition, *PTGS2 *mRNA has a very short half-life regulated by sequence-specific elements in the 3' untranslated region (UTR) of the gene [12,13].

We hypothesize that polymorphisms in this gene could influence the risk of breast cancer, through altering the levels of expression or activity of the PTGS2 enzyme. We have therefore genotyped five single-nucleotide polymorphisms (SNPs) in the *PTGS2 *gene in the breast cancer cases and controls nested within the Nurses' Health Study (NHS). One of these polymorphisms was statistically associated with decreased breast cancer risk, and we therefore sought to confirm this association in the breast cancer cases and controls nested within the Nurses' Health Study 2 (NHS2) and Harvard Women's Health Study (WHS).

## Materials and methods

The NHS nested breast cancer case-control study (cases, *n *= 1,270; controls, *n *= 1,762) is derived from 32,826 women who were free of diagnosed breast cancer at blood collection in 1989 and 1990, since when they were followed for incident disease until 31 May 2000. Medical records were used to confirm the diagnoses in women who reported a diagnosis of breast cancer on the biennial questionnaires. Control subjects were matched to cases on the basis of age, menopausal status, recent hormone replacement therapy, and blood-draw specific variables (such as date and time of day). The NHS2 breast cancer case-control study (cases, *n *= 317; controls, *n *= 634) is nested in a study of 29,611 women who were free of diagnosed breast cancer at the time of blood draw (between 1996 and 1999) and were followed for incident disease until 1 June 2003. The nested breast cancer case-control study in the WHS began in 1993, when 28,263 women provided blood samples and were followed for incident disease until 7 March 2000, with 702 cases and 703 controls. Cases and controls were selected for the NHS2 and WHS with the use of the same criteria as in the NHS. All three cohorts use similar questionnaires to collect covariate information, administered biennially. Detailed descriptions of these three cohorts have been published previously [14-16]. Informed consent was obtained from all participants, and the study was approved by the Institutional Review Board of the Brigham and Women's Hospital.

*PTGS2 *is a very small gene (less than 10 kilobase pairs from 5' UTR to 3' UTR), with only five known common polymorphisms; linkage disequilibrium between them is high (rs20417, rs5277, rs20432, rs5275, rs4648298 [17,18]; see Table 1 for more information). These five SNPs were genotyped by means of the 5' nuclease assay (TaqMan; Applied Biosystems, Foster City, CA, USA). The SNPs are located at base pairs 926, 3,050, 5,209, 8,473, and 9,850 on GenBank sequence D28235. rs20417 is in the 5' region, rs5277 is a synonymous polymorphism in exon 3, rs20432 is in the intron between exons 5 and 6, and both rs5275 and rs4648298 are in the 3' UTR of *PTGS2*. TaqMan primers, probes, and conditions for genotyping assays are available from the authors on request. All genotyping was done with laboratory personnel blinded to the case-control status of the samples and included quality-control samples (DNA from cohort participants, not part of the case-control study, repeated from 2 to 18 times across plates) for validation. Concordance for quality-control samples was 100%.

Statistical analysis was performed with SAS version 9.1 (SAS Institute, Cary, NC, USA). Haplotype estimation was performed with PROC HAPLOTYPE, tests for deviation from Hardy–Weinberg equilibrium were performed with PROC ALLELE, and haplotype-specific odds ratios and 95% confidence intervals were calculated with unconditional logistic regression with PROC LOGISTIC, controlling for age (at blood draw as a continuous variable), age at first birth (AFB), parity (nulliparous; one or two children and AFB 24 years or less; one or two children and AFB more than 24 years; more than two children and AFB 24 years or less; more than two children and AFB more than 24 years), menopausal status at diagnosis (premenopausal, postmenopausal, unknown), history of benign breast disease (yes/no), and family history of breast cancer (yes/no). Variables such as AFB, history of benign breast disease, or family history of breast cancer are updated in each questionnaire cycle, and the variable closest to the diagnosis (cases) or index date (controls) is used in these analyses. *Cis*-interactions, comparing associations with haplotypes to those observed with independent SNPs, were tested by comparing the model with the SNP genotype alone to the model with each haplotype carrying the risk allele by means of likelihood ratio testing. Interaction *p *values were calculated by likelihood ratio testing, comparing the model with main effects for each exposure (polymorphism and anti-inflammatory use) to the model including the cross-tabulation of the two exposures. Heterogeneity between cohorts was calculated with Cochran-Mantel-Haenszel statistics in PROC FREQ.

## Results

We did not detect any deviation from Hardy–Weinberg equilibrium among the controls in the NHS at any of the polymorphisms studied (data not shown). Only the rs5275 polymorphism in the 3' UTR of the *PTGS2 *gene showed an association with risk of breast cancer (Table 2) and was therefore subsequently genotyped in the NHS2 and WHS. There is almost complete linkage disequilibrium (Lewontin *D' *= 0.95) between the rs20417 polymorphism 5' of *PTGS2 *and rs5275 3' of *PTGS2*. Similarly, one *PTGS2 *haplotype carrying the variant allele of rs5275 was associated with a decrease in breast cancer risk. However, the global test for difference in haplotype frequencies between breast cancer cases and controls was not significant (*p *= 0.15; Table 3). Testing for *cis*-interaction confirmed that this haplotype was not more strongly associated with breast cancer risk than the rs5275 polymorphism alone. No difference in genotype distribution of rs5275 was observed between tumors positive or negative for sex steroid hormone receptors, no significant difference in risk was observed upon stratification by menopausal status, and no statistically significant interactions were detected between this polymorphism and use of aspirin or other non-steroidal anti-inflammatory drugs at baseline (evaluated only in the NHS; data not shown).

Similar genotype distributions were observed among both the NHS2 and WHS with respect to the NHS1 at rs5275. On pooling the three data sets (Table 4), no heterogeneity among the studies was detected (*p *= 0.12), and the pooled risk estimate shows a moderately statistically significant trend of decreasing risk with each variant rs5275 allele (odds ratio 0.91, 95% confidence interval 0.83 to 0.99, *p *= 0.02).

## Discussion

Variant expression or activity of PTGS2 could alter the risk of breast neoplasia, independently of exposure to aspirin. Prostaglandins stimulate cell migration and lead to proliferation and neovascularization [2]. Byproducts of the peroxidase activity of PTGS2 are free radicals, which are known mutagens [3-5]. Last, prostaglandin E_2 _induces aromatase, the main enzyme in the production of estrogen [19].

Recently Langsenlehner and colleagues published an association between the rs5275 SNP and breast cancer risk [20]. They observed a doubling in risk with the C/C genotype of this SNP in comparison with the T/T genotype. Interestingly, this polymorphism is not in Hardy–Weinberg equilibrium in either the case or the control group of the study presented by Langsenlehner and colleagues. Although the statistical significance of an inverse association between rs5275 and breast cancer risk among Caucasians (all three studies presented are more than 98% Caucasian in origin) as presented here requires replication, we can comfortably exclude a modest increase in risk associated with this genotype.

No *in vitro *or *in vivo *information regarding any putative change in function associated with this polymorphism is available. Given its location in the 3' UTR, the rs5275 polymorphism is a likely candidate to influence mRNA half-life, which is controlled by sequence-specific elements in this region of the mRNA [21]. It is possible that the rs5275 SNP is not a functional variant but a marker in linkage disequilibrium with variant(s) that alter breast cancer risk. Although linkage disequilibrium is strong across the *PTGS2 *gene, differences in the degree and extent of linkage disequilibrium between populations may explain variation in risk estimates from one study to another. Linkage between the rs5275 SNP and a causal variant would also explain why the strongest association with breast cancer risk is seen with this polymorphism and not the others. Examination of how the PTGS2 enzyme works in the breast, and in particular neoplastic breast cells, could shed light on its relation to breast cancer risk.

## Conclusion

Although polymorphisms, particularly the rs5275 polymorphism in the 3' UTR, of *PTGS2 *may be associated with a decrease in breast cancer risk, we provide strong evidence that it is not associated with an increased risk of breast cancer.

## Abbreviations

AFB = age at first birth; COX2 = cyclo-oxygenase 2; NHS = Nurses' Health Study; PTGS2 = prostaglandin endoperoxide synthase 2; SNP = single-nucleotide polymorphism; UTR = untranslated region; WHS = Harvard Women's Health Study.

## Competing interests

The authors declare that they have no competing interests.

## Authors' contributions

DGC performed analyses and prepared the manuscript. JB, SEH, and DJH were responsible for sample collection, editing the manuscript, and securing funding for the study. All authors read and approved the final manuscript.

## Figures and Tables

**Table 1:** Pairwise linkage disequilibrium between SNPs selected for study in *PTGS2*

SNP	PTGS2_20417	PTGS2_5277	PTGS2_5209	PTGS2_5275	PTGS2_4648298
PTGS2_20417	-	1	0.97	0.95	0.98
PTGS2_5277	0.03	-	1	1	0.84
PTGS2_5209	0.13	0.03	-	1	1
PTGS2_5275	0.1	0.06	0.11	-	1
PTGS2_4648298	0.01	0.002	0.02	0.01	-

Numbers above the diagonal are the Lewontin's *D'*, a measure of linkage disequilibrium; numbers under the diagonal are the correlation coefficient *r*. PTGS2, prostaglandin endoperoxide synthase 2; SNP, single-nucleotide polymorphism.

**Table 2:** Association between *PTGS2 *genotypes and breast cancer risk in the Nurses' Health Study

SNP	Genotype	Cases (percentage)	Controls (percentage)	OR (95% CI)^a^
PTGS2_20417	G/G	865 (69.6)	1,185 (69.1)	1.00 (reference)
	G/C	336 (27.0)	485 (28.3)	0.93 (0.78–1.10)
	C/C	42 (3.4)	45 (2.6)	1.28 (0.82–2.02)
				*p *trend = 0.94
PTGS2_5277	G/G	875 (71.0)	1,217 (71.0)	1.00 (reference)
	G/C	322 (26.1)	464 (27.1)	1.00 (0.84–1.19)
	C/C	35 (2.8)	34 (2.0)	1.35 (0.81–2.26)
				*p *trend = 0.55
PTGS2_5209	T/T	834 (69.4)	1,171 (69.5)	1.00 (reference)
	T/G	330 (27.5)	472 (28.0)	0.97 (0.82–1.16)
	G/G	38 (3.2)	43 (2.6)	1.24 (0.77–2.00)
				*p *trend = 0.81
PTGS2_5275	T/T	541 (43.3)	699 (40.6)	1.00 (reference)
	T/C	567 (45.4)	808 (47.0)	0.88 (0.75–1.04)
	C/C	141 (11.3)	213 (12.4)	0.80 (0.62–1.03)
				*p *trend = 0.05
PTGS2_4648298	A/A	1,180 (95.4)	1,654 (96.3)	1.00 (reference)
	A/G + G/G	57 (4.6)	63 (3.7)	1.29 (0.88–1.88)

^a^Unconditional logistic regression controlled for age, BMI at age 18 years, menopausal status at diagnosis, history of benign breast disease, and family history of breast cancer. CI, confidence interval; PTGS2, prostaglandin endoperoxide synthase 2; OR, odds ratio; SNP, single-nucleotide polymorphism.

**Table 3:** PTGS2 haplotypes and breast cancer risk in the Nurses' Health Study

Haplotype	Cases (percentage)	Controls (percentage)	OR (95% CI)^a^
z1 G-G-T-T-A	1,250 (49.4)	1,691 (48.2)	1.00 (reference)
z2 G-G-T-C-A	435 (17.2)	667 (19.0)	0.84 (0.72–0.98)
z3 G-C-T-T-A	403 (15.9)	544 (15.5)	0.99 (0.85–1.17)
z4 C-G-G-C-A	358 (14.1)	507 (14.5)	0.92 (0.79–1.09)
z5 C-G-G-C-G	53 (2.1)	63 (1.8)	1.16 (0.78–1.72)
			Global *p *= 0.15

^a^Unconditional logistic regression controlled for age, BMI at age 18 years, menopausal status at diagnosis, history of benign breast disease, and family history of breast cancer. CI, confidence interval; OR, odds ratio; PTGS2, prostaglandin endoperoxide synthase 2.

**Table 4:** *PTGS2 *rs5275 genotype and breast cancer risk within the NHS1, NHS2, WHS, and pooled data

Study	Genotype	Cases (percentage)	Controls (percentage)	OR (95% CI)^a^
NHS1	T/T	541 (43.3)	699 (40.6)	1.00 (reference)
	T/C	567 (45.4)	808 (47.0)	0.89 (0.75–1.04)
	C/C	141 (11.3)	213 (12.4)	0.80 (0.62–1.03)
				*p *trend = 0.05
NHS2	T/T	140 (46.5)	270 (44.3)	1.00 (reference)
	T/C	131 (43.5)	259 (42.5)	0.97 (0.72–1.31)
	C/C	30 (10.0)	81 (13.3)	0.72 (0.45–1.15)
				*p *trend = 0.26
WHS	T/T	281 (43.6)	278 (42.7)	1.00 (reference)
	T/C	296 (46.0)	294 (45.2)	0.99 (0.78–1.26)
	C/C	67 (10.4)	79 (12.1)	0.86 (0.58–1.25)
				*p *trend = 0.53
Pooled	T/T	962 (43.8)	1,246 (41.8)	1.00 (reference)
	T/C	994 (45.3)	1,361 (45.7)	0.93 (0.82–1.05)
	C/C	238 (10.8)	373 (12.5)	0.80 (0.66–0.97)
				*p *trend = 0.02

^a^Unconditional logistic regression controlled for age, age at first birth and parity, menopausal status at diagnosis, history of benign breast disease, and family history of breast cancer. CI, confidence interval; NHS, Nurses' Health Study; OR, odds ratio; PTGS2, prostaglandin endoperoxide synthase 2; WHS, Harvard Women's Health Study.
